# Predisposing factors for symptoms of anxiety, depression, and insomnia in university students

**DOI:** 10.1590/0034-7167-2023-0387

**Published:** 2024-12-16

**Authors:** Júlia Couto de Oliveira, Vanessa Ribeiro Neves, Juliana Garcia Céspedes, Vânia D’Almeida, Meiry Fernanda Pinto Okuno, Laís Lira Figueiredo, Ana Rafaela de Brito Cerqueira, Anderson da Silva Rosa

**Affiliations:** I Universidade Federal de São Paulo. São Paulo, São Paulo, Brazil

**Keywords:** Anxiety, Depression, Insomnia, Mental Health, Students., Ansiedad, Depresión, Estudiantes, Insomnio, Salud mental.

## Abstract

**Objectives:**

to characterize the sociodemographic and psychological aspects of university students who sought psychiatric care at a Student Support Center of a Federal University and to analyze associations between mental health issues and predisposing factors.

**Methods:**

a retrospective analysis of 103 medical records was conducted. The statistical analysis consisted of two steps: a descriptive analysis and a predictive analysis using the Logistic Regression Model.

**Results:**

the majority of the students were female. Symptoms of anxiety, depression, and insomnia were the main reasons for seeking care. Students who reported having emotional difficulties that negatively impacted their studies and those who had undergone some form of health treatment showed a higher probability of experiencing anxiety symptoms. Notable correlations were found between anxiety and emotional difficulties, depression and diarrhea, and insomnia and a sedentary lifestyle.

**Conclusions:**

symptoms of anxiety, depression, and insomnia led students to seek psychiatric care at the university. Understanding the predisposing factors for mental health issues in university students can inform care strategies and promote academic success.

## RESUMEN

**Objetivos:**

caracterizar los aspectos sociodemográficos y psicológicos de los estudiantes universitarios que buscaron atención psiquiátrica en un Núcleo de Apoyo Estudiantil de una Universidad Federal y analizar las asociaciones entre las alteraciones de salud mental y los factores predisponentes.

**Métodos:**

se realizó un análisis retrospectivo de 103 historiales médicos. El análisis estadístico consistió en dos etapas: un análisis descriptivo y un análisis predictivo utilizando el Modelo de Regresión Logística.

**Resultados:**

la mayoría de los estudiantes eran mujeres. Los síntomas de ansiedad, depresión e insomnio fueron las principales razones para buscar atención. Los estudiantes que informaron tener dificultades emocionales que afectaron negativamente sus estudios y aquellos que habían recibido algún tipo de tratamiento de salud mostraron una mayor probabilidad de experimentar síntomas de ansiedad. Se encontraron correlaciones significativas entre ansiedad y dificultades emocionales, depresión y diarrea, e insomnio y un estilo de vida sedentario.

**Conclusiones:**

los síntomas de ansiedad, depresión e insomnio llevaron a los estudiantes a buscar atención psiquiátrica en la universidad. Conocer los factores predisponentes de los problemas de salud mental en los estudiantes universitarios puede fundamentar estrategias de cuidado y promover el éxito académico.

## INTRODUCTION

The university student is increasingly vulnerable to mental health and sleep problems, being three times more likely to develop psychological illnesses when compared to the non-university population, which can result in low academic performance and even dropout^(1)^. Studies show that mental illness among undergraduate students is a global problem^(2-4)^.

The transition to academic life involves intense physical and psychosocial changes in the university population, favoring or enhancing the development of mental disorders^(5)^. Psychological distress during college is determined by academic and sociodemographic aspects, predisposing psychological factors, health habits, and interpersonal relationships^(6)^.

Risk factors related to academic issues include poor expectations about future careers and the desire to drop out of courses. Regarding students’ health habits, sedentary behavior and drug use are noteworthy. Social aspects reveal discrimination related to race/ethnicity, gender, and class. Psychological factors include negative feelings, low self-esteem, and not sharing problems. In interpersonal relationships, there is a lack of social support, difficulty in relationships, and not feeling a sense of belonging to the academic environment^(6)^.

Depression and anxiety are the most recurrent mental disorders among university students, often associated with each other. The main symptoms presented are fear, sleep disturbances, palpitations, fatigue, decreased pleasure in activities, weight changes, and difficulty concentrating^(7)^. A study conducted with health students at the Federal University of Petrolina, Pernambuco, showed that out of 410 students, 53.3% showed signs of mental distress; among the disorders presented, anxiety ranked first, followed by stress, and finally depression^(8)^. The consequences of these mental disorders include drug abuse, high-risk behavior, low academic performance, and university dropout, potentially leading to suicide attempts^(8-13)^.

The National Association of Federal Institutions (in Portuguese ANDIFES) conducted the 5th National Survey on Socioeconomic and Cultural Profile in 2018, which highlighted data related to the mental health of university students in federal institutions in Brazil. Six out of every ten students reported feeling anxious during their undergraduate studies^(14)^. Additionally, 32.4% of students reported undergoing psychological treatment, 9.8% had used psychiatric medication, and 6.5% were using it at the time of the survey. There was a significant increase among university students who reported death ideation (10.8%), as well as suicidal thoughts (8.8%), compared to findings from previous surveys conducted by the same institution^(14)^.

The present study is justified by the importance of identifying factors that are correlated with the development of mental illnesses among university students, enabling an epidemiological analysis of the problem at a Federal University. It also aims to contribute to the creation and implementation of actions that can reduce these factors, aiming to improve the quality of life of students.

## OBJECTIVES

To characterize the sociodemographic and psychological aspects of university students who sought psychiatric care at a Student Support Center of a Federal University and to analyze associations between mental health disorders and predisposing factors.

## METHODS

### Ethical aspects

The research was approved by the Research Ethics Committee of UNIFESP, ensuring compliance with Resolution 466/2012 of the National Health Council. Data collection was conducted confidentially and without the disclosure of individualized personal information, as guaranteed by the Data and Records Use Commitment Agreement signed by the researchers. Therefore, the signing of the Free and Informed Consent Form was not applicable to this study.

### Design, period, and location of the study

This study was developed following the guidelines of the EQUATOR network, using the Strengthening the Reporting of Observational Studies in Epidemiology (STROBE) instrument. A retrospective review was conducted on the medical records and initial clinical evaluation forms of students who sought psychiatric care at the Student Support Center of a federal university in the state of São Paulo. The campus where the research was conducted offers undergraduate courses in Nursing, Medicine, Biomedical Sciences, Speech Therapy, and Technology. Data collection took place between September 2020 and February 2021.

### Population, sample, and selection criteria

The initial sample, established for convenience, consisted of 127 medical records of students who sought psychiatric care between 2017 and 2019. The inclusion criteria were having an Initial Clinical Evaluation form and consultation records available for analysis. Based on these criteria, the study sample comprised 103 medical records.

The Initial Clinical Evaluation form consists of a semi-structured self-administered questionnaire provided to students seeking healthcare. It includes questions related to sociodemographic profile, health history, habits, and behaviors. Data on mental health disorders presented by the students during psychiatric consultations were collected through the retrospective review of medical records.

### Data Analysis

Statistical analysis was conducted in two stages: a descriptive analysis and a predictive analysis. The descriptive analysis aimed to summarize and explore the dataset using absolute and relative frequencies, means, standard deviations, medians, as well as range (minimum and maximum). The predictive analysis, on the other hand, allows for the identification of patterns among variables that indicate the likelihood of a university student developing mental health issues. In other words, by identifying common characteristics among the students who were attended, it is possible to establish prevention policies for those more susceptible to symptoms of anxiety, depression, and insomnia. The primary objective of predictive analysis is to achieve a better assessment of what may happen in the future.

This model enabled the identification, based on the completion of the Initial Clinical Evaluation form, of the student profile most likely to present symptoms of mental disorders. After adjusting the logistic regression model, the Hosmer-Lemeshow test was performed, with a significance level of 5%, to define the accuracy of the model used in the analyses^(15)^.

The models were adjusted considering a significance level of 10% with all variables. Then, the backward variable selection method was used to remove the least performing variables from the model, comparing the Akaike Information Criterion (AIC) statistic with the complete model. The method concludes when, in a given iteration, no variable is removed, and the remaining variables are then defined by the final model.

## RESULTS

Regarding the sociodemographic profile of the students who sought psychiatric care, the majority are female (62; 60.19%), followed by male (41; 39.81%), are in the age range of 18 to 20 years (56; 54.37%), self-identify as white (69; 66.99%), are single (102; 99.03%), do not have medical insurance (57; 55.34%), do not live in student dormitories (75; 72.82%), are proportionally more represented among students of medicine (47; 45.63%) and nursing (27; 26.21%), and mostly enrolled between the years 2015 and 2017 (72; 69.91%). Table 1 presents the health history of these students, and Table 2 presents their habits and behaviors.

**Table 1:** Distribution of students according to health history, São Paulo, São Paulo, Brazil, 2020-2021 (N=103)

Health History	Yes (n (%))	No (n (%))	Total (N (%))
Undergoes any health treatment	23 (22.33%)	80 (77.67%)	103 (100%)
Uses any medication	38 (36.89%)	65 (63.11%)	103 (100%)
Often has diarrhea	10 (9.71%)	93 (90.2%)	103 (100%)
Often has headaches	47 (45.63%)	56 (54.37%)	103 (100%)

**Table 2:** Distribution of students according to habits and behaviors, São Paulo, São Paulo, Brazil, 2020-2021 (N=103)

Habits and Behaviors	n (%)
Has sought psychological care	
Yes	53 (51.4%)
No	50 (48.54%)
Has sought psychiatric care	
No	78 (75.73%)
Yes	25 (24.27%)
Takes any psychiatric medication	
No	89 (86.41%)
Yes	14 (13.59%)
Has had any emotional difficulty that affected studies	
Yes	53 (51.46%)
No	50 (48.54%)
Engages in physical activities	
Daily or more than 3 times a week	25 (24.27%)
Less than 3 times a week	42 (40.78%)
Never	36 (34.95%)
Sleeps well and feels rested	
Always or Usually	20 (19.42%)
Sometimes	51 (49.51%)
Almost never or rarely	32 (31.07%)
Uses drugs	
Yes	68 (66.02%)
No	33 (32.04%)
Not reported	2 (1.94%)
Total	103 (100%)

As shown in Table 2, 51.4% of students have sought psychological care, and 51.46% reported having experienced emotional difficulties that affected their studies. Additionally, 66.02% of students indicated drug use, and 31.07% stated that they almost never or rarely sleep well and feel rested.

**Table 3:** Final adjusted model for the Anxiety variable, São Paulo, São Paulo, Brazil, 2020-2021 (N=103)

Explanatory Variables	Coefficient	*p* value
Intercept	0.006	0.985
Undergoes any health treatment	1.074	0.076^*^
Uses any medication	-1.216	0.029^**^
Has any skin problems	0.532	0.369
Has experienced weight changes	-0.744	0.110
Has had any emotional difficulty that affected studies	0.981	0.056^*^

* significant at 10%;

** significant at 5%.

**Table 4:** Final adjusted model for the Depression variable, São Paulo, São Paulo, Brazil, 2020-2021 (N=103)

Explanatory Variables	Coefficient	*p* value
Intercept	-0.096	0.758
Has heart problems	17.181	0.992
Often has diarrhea	1.919	0.025^**^
Has intestinal problems	0.685	0.140
Has had any emotional difficulty that affected studies	-0.974	0.029^**^

* significant at 10%;

** significant at 5%.

**Table 5:** Final adjusted model for the Insomnia variable, São Paulo, São Paulo, Brazil, 2020-2021 (N=103)

Explanatory Variables	Coefficient	*p* value
Intercept	-1.456	0.006^**^
Has had previous psychological treatment	1.292	0.032^**^
Uses any psychiatric medication	-2.401	0.041^**^
Often has diarrhea	1.219	0.041^**^
Often has headaches	1.810	0.002^**^
Has had any emotional difficulty that affected studies	-1.309	0.036^**^
Does not engage in physical activity	0.977	0.089^*^
Engages in physical activity > 3 times a week	-0.544	0.470
Engages in physical activity daily	0.615	0.487

* significant at 10%;

** significant at 5%.

Among the mental health issues described in the medical records that motivated students to seek psychiatric care, anxiety was the most prominent (53; 51.45%), followed by depression (48; 46.6%) and insomnia (40; 38.83%).

### Logistic Regression Model

In this study, three random variables were analyzed independently, with responses classified as Y=1 if the individual presented the characteristic and Y=0 if not. These variables were related to the mental health issues students presented when seeking psychiatric care: anxiety, depression, and insomnia.

Each of these variables (anxiety, depression, insomnia) was related to the following explanatory variables: course; year of enrollment; age; sex; race/ethnicity; medical insurance; health treatment; medication use; family history; whether they have skin problems; whether they have heart problems; whether they have stomach problems; whether they often have diarrhea; whether they often have constipation; whether they experience weight changes; whether they often have headaches; whether they have had seizures; prior psychological care; psychiatric care; psychiatric medication; psychiatric diagnosis; emotional difficulty; physical activity; whether they sleep well and feel rested; drug use; and caffeine intake, as shown in Tables 3, 4, and 5.

It is observed that students who reported having experienced some emotional difficulty that affected their studies (p-value = 0.056) have a higher probability of developing anxiety during their undergraduate studies compared to those who denied such emotional difficulties. Those who have undergone any type of health treatment are more likely (p-value = 0.076) to develop anxiety. On the other hand, students who use some type of medication are less likely (p = 0.029) to experience anxiety.

Students who often experience diarrhea (p = 0.025) have a higher probability of developing depression compared to those who denied having this physical symptom. On the other hand, students who reported having experienced some emotional difficulty that affected their studies (p = 0.029) are less likely to develop depression.

Diarrhea (p = 0.041) and headaches (p = 0.002) are physical symptoms associated with insomnia. Students experiencing these symptoms have a higher probability of having insomnia, while those who use psychiatric medications (p = 0.041) or have emotional difficulties (p = 0.036) are less likely to experience insomnia.

Additionally, university students who reported having undergone previous psychological treatment (p = 0.032) have a higher probability of developing insomnia compared to those who denied it. Regarding physical activity, students who do not exercise (p = 0.089) are more likely to experience insomnia compared to those who engage in physical activity at least three times a week.

## DISCUSSION

The present study allowed us to characterize the sociodemographic and psychological profiles of undergraduate health students who sought psychiatric care and to analyze the predisposing factors for mental health disorders.

The female gender was found to be more prevalent compared to the male gender, corroborating findings in the literature that reveal a higher incidence of mental disorders among women, such as depression, anxiety, and suicide attempts^(16,17)^. According to the World Health Organization (WHO), the prevalence of anxiety among women in the Americas is 7.7%^(18)^. Gender violence, combined with the accumulation of multiple roles, such as academic, socio-family, and economic responsibilities, are factors that predispose women to mental illness^(19)^.

Health students engage in university life more intensely when they come into contact with diseases, death, and the suffering of patients and their families^(20)^. In this study, the Medicine and Nursing courses had, proportionally, higher numbers of students seeking psychiatric care compared to other undergraduate courses such as Biomedical Sciences, Speech Therapy, and Technology. This finding is not conclusive since the Nursing and Medicine courses offer a greater number of annual spots and, consequently, have a higher percentage of students on the study campus. However, research indicates that mental illness can result from greater contact with clinical practice and the hospital environment^(21,22)^.

Drug use was reported by the majority of university students (68; 66.02%), corroborating literature that shows high rates of alcohol use in universities^(23)^. Such consumption is considered a coping strategy for various academic life conditions that create vulnerability to mental disorders, such as academic demands and concerns about entering the job market^(20)^. Mental illness can also become a predisposing factor for alcohol abuse, which is correlated with the use of illicit drugs, high cigarette consumption, and increased risky behaviors, such as unprotected sex and driving under the influence of alcohol^(24,25)^.

A survey conducted by the WHO in 2016 showed that 26.4 million people worldwide suffer from anxiety disorders^(18)^. This disorder was the main complaint among students who sought psychiatric care at the university. Out of 103 students, 53 (51.45%) reported feeling anxious, consistent with other studies showing high levels of anxiety among university students^(26,27)^.

Emotional difficulties were present in the majority of university students; about 53 (51.46%) students reported having some emotional difficulty that negatively affected their studies. Data from the 5th National Survey on Socioeconomic and Cultural Profile conducted by ANDIFES in 2018 corroborate the results presented: 83.5% of students in different federal institutions reported having experienced emotional difficulties that negatively affected their academic life, such as anxiety, sadness, shyness, panic, and insomnia^(14)^.

On the other hand, in the present study, emotional difficulty was found to be a protective factor against depression and insomnia. This finding contradicts the literature, which shows that emotional difficulty can interfere with academic performance and quality of life, potentially leading to university dropout, stress, anger, sadness, and discouragement-situations that predispose individuals to mental illness^(28,29)^.

Furthermore, students who reported having had such emotional difficulties are more likely to develop anxiety. Emotional exhaustion, resulting from the depersonalization of university students through excessive demands, intense workload, a high number of evaluations, as well as interpersonal conflicts with peers and professors, is a facilitator for the development of anxiety^(30)^.

Stress is significantly present among university students^(31)^. One of the main protective physiological responses of the human body to stress is the release of the glucocorticoid hormone cortisol by the Hypothalamic-Pituitary-Adrenal axis of the neuroendocrine system^(32,33)^. However, elevated cortisol release over a long period can result in significant metabolic changes, such as type 2 diabetes mellitus, hypertension, and obesity^(33)^. In addition, chronic stress can lead to the onset of Burnout syndrome, characterized by physical and mental exhaustion^(34)^.

In this study, students who reported undergoing some form of health treatment were more likely to experience anxiety. Physical illness among university students is also a predisposing factor for the presence of mental disorders^(35,36)^.

According to ANDIFES, about 23.2% of university students in Federal Institutions of Higher Education seek medical services for specific treatments, and 32.4% of students have sought psychological care^(14)^. These results are consistent with the present study, in which 22.33% of the university students attended were undergoing some form of health treatment. Regarding psychological treatment, a higher rate was observed, with about 51.4% of students reporting seeking this type of care.

Previous psychiatric care was found in 24.27% of students, and psychotropic drug use in 13.59%, showing a higher rate compared to other students from federal institutions^(14)^.

Regarding medication use, 38 (36.8%) students reported using some medication, showing a lower rate compared to a study conducted at a university in the Northern Plateau of Santa Catarina, where 234 students (57.2%) were using some medication^(37)^. Additionally, according to the present study, taking medication is a protective factor against the development of anxiety, agreeing with other studies that show that seeking health care, self-perception, and emotional rebalancing reduce predisposing factors to mental illness^(38,39)^. However, the indiscriminate use of medications among university students can lead to adverse reactions, intoxication, and dependence^(39)^.

Depressive symptoms rank as the second most common reason for seeking psychiatric care. This finding aligns with studies that identify dissatisfaction with the course, competition in the academic environment, high demands during evaluations, reduced family support, insufficient sleep, loneliness, and lack of leisure as predisposing factors for the development of depression^(11,40)^. Consequently, there is a decrease in memory capacity, motivation, and reasoning, which interferes with academic performance^(40)^. Early diagnosis and treatment are of utmost importance, as they prevent risky behaviors and suicidal ideation among university students^(40)^.

The association between gastrointestinal symptoms and mental disorders was also a result of this research. Students who reported having diarrhea are more likely to develop symptoms of depression and insomnia. This finding is consistent with the literature, which shows that stress interferes with the functioning of the human body’s systems, compromising cellular components and triggering diseases such as Irritable Bowel Syndrome^(41)^. Furthermore, an integrative review of the literature related to the Pittsburgh Sleep Quality Index in medical students showed a high prevalence of poor sleep quality among students with Irritable Bowel Syndrome^(42)^.

Headaches were present in almost half of the students who sought care at the university. These students are more likely to develop insomnia, in agreement with the literature^(43)^. Sleep plays an important neuronal role, affecting cognitive functioning^(44)^. Dysfunction in the sleep-wake cycle leads to poor academic performance, interfering with the professional development of students^(45)^. Thus, insomnia was the third most reported issue in psychiatric care, corroborating findings from other studies^(46,47)^.

The practice of physical exercise proved to be a protective factor against insomnia. Students who do not engage in physical activity are more likely to develop insomnia compared to those who exercise at least three times a week. Exercise puts the body into a catabolic process that needs to be followed by an anabolic process, resulting in greater relaxation and a desire to sleep^(48)^. In addition, physical exercise provides better emotional regulation, reducing stress and improving sleep quality^(46)^.

### Study limitations

The research was conducted on a campus that only offers health-related courses, which does not allow for determining patterns of mental illness across different types of courses and student profiles. Furthermore, the evaluated instrument was designed to collect general information from students to assess overall needs and therefore does not delve deeply into issues that would be relevant for understanding mental health illness processes.

### Contributions to the Health Field

The results of this study can provide a basis for strategies to improve the quality of life and academic success of university students.

## CONCLUSIONS

Female students comprised the majority of those seeking psychiatric care at a university student service center. Symptoms of anxiety, depression, and insomnia were the main reasons for seeking care. Inferential analysis found a higher statistical probability for anxiety symptoms among students who reported emotional difficulties and prior health treatment, a higher probability of depression symptoms among students who reported having diarrhea, and a higher probability of insomnia among students who reported diarrhea, headaches, and did not engage in physical activity. University life coincides with the transition to adulthood and increased responsibilities for most students. Expectations for academic performance and professional success are created, along with the influence of biographical factors such as gender, race/ethnicity, and social class, which can affect mental health and illness processes.
