# Supplementary material for: Predisposing factors for symptoms of anxiety, depression, and insomnia in university students
Source: Rev Bras Enferm. 2024 Dec 16;77(6):e20230387. doi: 10.1590/0034-7167-2023-0387 (PMC11654530; doi:10.1590/0034-7167-2023-0387)
Supplement: Translation

# Translation: Fatores predisponentes para sintomas de ansiedade, depressão e insônia em estudantes universitários

**DOI:** 10.1590/0034-7167-2023-0387pt

**Authors:** Júlia Couto de Oliveira, Vanessa Ribeiro Neves, Juliana Garcia Céspedes, Vânia D’Almeida, Meiry Fernanda Pinto Okuno, Laís Lira Figueiredo, Ana Rafaela de Brito Cerqueira, Anderson da Silva Rosa

**Affiliations:** I Universidade Federal de São Paulo. São Paulo, São Paulo, Brasil

**Keywords:** Ansiedade, Depressão, Estudantes, Insônia, Saúde Mental.

## Abstract

**Objetivos:**

caracterizar aspectos sociodemográficos e psicológicos de estudantes universitários que buscaram atendimento psiquiátrico em um Núcleo de Apoio Estudantil de uma Universidade Federal e analisar associações entre alterações de saúde mental e fatores predisponentes.

**Método:**

análise retrospectiva de 103 prontuários. A análise estatística consistiu em duas etapas: uma descritiva e uma preditiva pelo Modelo de Regressão Logística.

**Resultados:**

a maioria dos estudantes é do gênero feminino. Sintomas de ansiedade, depressão e insônia foram os principais motivos para a procura de atendimento. Estudantes que afirmaram ter dificuldades emocionais prejudiciais aos estudos e os que realizaram algum tratamento para saúde apresentaram maior probabilidade de apresentar sintomas de ansiedade. Destacaram-se correlações entre ansiedade e dificuldades emocionais; depressão e diarreia; e insônia e sedentarismo.

**Conclusões:**

sintomas de ansiedade, depressão e insônia levaram estudantes ao atendimento psiquiátrico na universidade. Conhecer fatores predisponentes para adoecimento em saúde mental de universitários pode fundamentar estratégias de cuidado e favorecer o sucesso acadêmico.

## INTRODUÇÃO

O estudante universitário está cada vez mais vulnerável a problemas de saúde mental e sono, apresentando três vezes mais chances de adoecimento psíquico quando comparado à população não universitária, podendo apresentar baixo rendimento acadêmico e até evadir da universidade^(1)^. Dessa maneira, estudos mostram que o adoecimento mental entre estudantes de graduação é um problema mundial^(2-4)^.

A transição para a vida acadêmica envolve intensas mudanças físicas e psicossociais na população universitária, favorecendo ou potencializando o desenvolvimento de transtornos mentais^(5)^. O sofrimento psíquico durante a graduação é determinado por aspectos acadêmicos e sociodemográficos, fatores psicológicos predisponentes, hábitos de saúde e relações interpessoais^(6)^.

Entre os fatores de risco relacionados às questões acadêmicas estão expectativas ruins sobre o futuro profissional e o desejo de abandonar o curso. Já em relação aos hábitos de saúde do estudante, destacam-se o sedentarismo e o uso de drogas. Em aspectos sociais, evidencia-se a discriminação em relação à raça/etnia, gênero e classe. Como fatores psicológicos, destacam-se sentimentos negativos, baixa autoestima e o não compartilhamento de problemas. Nas relações interpessoais, há fragilidade do apoio social, dificuldade de relacionamento e o fato de não se sentir pertencente ao ambiente acadêmico^(6)^.

Depressão e ansiedade são os transtornos mentais mais recorrentes entre estudantes universitários, estando habitualmente associados entre si. Os principais sintomas apresentados são medo, distúrbios do sono, palpitações, fadiga, diminuição do prazer nas atividades, alterações de peso e dificuldade de concentração^(7)^. Um estudo realizado com estudantes da área da saúde na Universidade Federal de Petrolina, Pernambuco, mostrou que, de 410 estudantes, 53,3% apresentavam indícios de sofrimento mental; entre os transtornos apresentados, em primeiro lugar esteve a ansiedade, posteriormente o estresse e, por fim, a depressão^(8)^. As consequências da presença desses transtornos mentais são abuso de drogas, comportamento de alto risco, baixo rendimento acadêmico e evasão da universidade, podendo chegar até à tentativa de suicídio^(8-13)^.

A Associação Nacional de Instituições Federais (ANDIFES) realizou a V Pesquisa Nacional de Perfil Socioeconômico e Cultural de 2018, que evidenciou dados em relação à saúde mental de estudantes universitários das instituições federais do Brasil. A cada 10 estudantes, seis referiram sentir-se ansiosos durante a graduação^(14)^. Além disso, 32,4% dos estudantes afirmaram estar realizando tratamento psicológico, 9,8% utilizaram medicação psiquiátrica e 6,5% utilizavam no momento da pesquisa. Houve um aumento significativo entre universitários que apresentaram idealização de morte (10,8%), assim como pensamentos suicidas (8,8%), quando esses achados foram comparados aos de pesquisas anteriores realizadas pela mesma instituição^(14)^.

O presente estudo justifica-se pela importância de identificar fatores que estejam correlacionados ao desenvolvimento de doenças mentais em estudantes universitários, possibilitando uma análise epidemiológica sobre o problema em uma Universidade Federal, além de contribuir para a criação e implementação de ações que sejam capazes de reduzir esses fatores, visando à melhoria na qualidade de vida dos estudantes.

## OBJETIVOS

Caracterizar aspectos sociodemográficos e psicológicos de estudantes universitários que buscaram atendimento psiquiátrico em um Núcleo de Apoio Estudantil de uma Universidade Federal e analisar associações entre alterações de saúde mental e fatores predisponentes.

## MÉTODOS

### Aspectos éticos

A pesquisa foi aprovada pelo Comitê de Ética em Pesquisa da UNIFESP, garantindo o cumprimento da Resolução 466/2012 do Conselho Nacional de Saúde. Por meio do Termo de Compromisso para Utilização de Dados e Prontuários, assinado pelos pesquisadores, a coleta aconteceu de forma sigilosa e sem divulgação de informações pessoais individualizadas, de modo que não se aplica ao presente estudo a assinatura do Termo de Consentimento Livre e Esclarecido.

### Desenho, período e local do estudo

Este estudo foi elaborado a partir das diretrizes da rede EQUATOR, por meio do instrumento *Strengthening the Reporting of Observational Studies in Epidemiology* (STROBE). Foi realizada a leitura retrospectiva de prontuários e da ficha de avaliação clínica inicial dos estudantes que procuraram atendimento psiquiátrico no Núcleo de Apoio Estudantil de uma universidade federal no estado de São Paulo. O campus onde a pesquisa foi realizada oferece os cursos de graduação em Enfermagem, Medicina, Biomedicina, Fonoaudiologia e Tecnologias. A coleta de dados foi realizada entre setembro de 2020 e fevereiro de 2021.

### População, amostra e critérios de seleção

A amostragem inicial, estabelecida por conveniência, foi composta por 127 prontuários de estudantes que procuraram atendimento psiquiátrico entre os anos de 2017 e 2019. Os critérios de inclusão na amostra foram ter a ficha de Avaliação Clínica Inicial e o prontuário das consultas disponíveis para análise. Por meio desses critérios, a amostra do estudo conteve 103 prontuários.

A ficha de Avaliação Clínica Inicial é constituída por um questionário semiestruturado de autopreenchimento, disponibilizado ao estudante que procura o atendimento de saúde. Engloba questões relacionadas ao perfil sociodemográfico, histórico de saúde, hábitos e comportamentos. Os dados sobre as alterações de saúde mental apresentadas pelos estudantes nas consultas com o médico psiquiatra foram coletados durante a leitura retrospectiva dos prontuários.

### Análise dos dados

A análise estatística foi realizada em duas etapas: uma análise descritiva e uma preditiva. A análise descritiva tem como objetivo resumir e explorar o conjunto de dados por meio de frequências absolutas, relativas, médias, desvio-padrão e medianas, bem como variação (mínimo e máximo). Já a análise preditiva permite identificar um padrão de variáveis que identifique a probabilidade de um estudante da universidade vir a apresentar alterações de saúde mental. Ou seja, na vigência de características comuns entre os estudantes atendidos, é possível estabelecer políticas de prevenção para aqueles mais suscetíveis a apresentar sintomas de ansiedade, depressão e insônia. O objetivo principal de uma análise preditiva é obter uma melhor avaliação do que poderá acontecer no futuro.

Este modelo permitiu identificar, a partir do preenchimento da ficha de Avaliação Clínica Inicial, qual era o perfil de estudante que teria maior probabilidade de apresentar sintomas de transtornos mentais. Após o ajuste do modelo de regressão logística, foi realizado o teste de Hosmer-Lemeshow, com nível de significância de 5%, para definir a acurácia do modelo utilizado nas análises^(15)^.

Os modelos foram ajustados considerando o nível de significância de 10% com todas as variáveis e, em seguida, utilizou-se o método de seleção de variáveis *backward* para eliminar do modelo as variáveis de pior desempenho, comparando a estatística AIC (*Akaike Information Criterion*) com o modelo completo. O método se encerra quando, em uma determinada rodada, não se retira nenhuma variável, sendo então as variáveis restantes definidas pelo modelo final.

## RESULTADOS

Em relação ao perfil sociodemográfico dos estudantes que procuraram atendimento psiquiátrico, a maioria é do gênero feminino (62; 60,19%), seguida do gênero masculino (41; 39,81%), está na faixa etária de 18 a 20 anos (56; 54,37%), é autodeclarada da cor branca (69; 66,99%), solteira (102; 99,03%), não possui convênio médico (57; 55,34%), não reside em repúblicas estudantis (75; 72,82%), é proporcionalmente maior entre alunos dos cursos de medicina (47; 45,63%) e enfermagem (27; 26,21%) e tem ano de ingresso prevalente entre 2015 e 2017 (72; 69,91%). A Tabela 1 apresenta o histórico de saúde desses estudantes e a Tabela 2 apresenta seus hábitos e comportamentos.

**Tabela 1:** Distribuição de estudantes segundo histórico de saúde, São Paulo, São Paulo, Brasil, 2020-2021 (N=103)

Histórico de saúde	Sim (n (%))	Não (n (%))	Total (N (%))
Faz algum tratamento de saúde	23 (22,33%)	80 (77,67%)	103 (100%)
Faz uso de algum medicamento	38 (36,89%)	65 (63,11%)	103 (100%)
Costuma ter diarreia	10 (9,71%)	93 (90,2%)	103 (100%)
Costuma ter dor de cabeça	47 (45,63%)	56 (54,37%)	103 (100%)

**Tabela 2:** Distribuição de estudantes segundo hábitos e comportamentos, São Paulo, São Paulo, Brasil, 2020-2021 (N=103)

Hábitos e comportamentos	n (%)
Já procurou atendimento psicológico	
Sim	53 (51,4%)
Não	50 (48,54%)
Já procurou atendimento psiquiátrico	
Não	78 (75,73%)
Sim	25 (24,27%)
Toma algum medicamento psiquiátrico	
Não	89 (86,41%)
Sim	14 (13,59%)
Já teve alguma dificuldade emocional que prejudicou os estudos	
Sim	53 (51,46%)
Não	50 (48,54%)
Realiza atividades físicas	
Diariamente ou mais que 3x na semana	25 (24,27%)
Menos que 3x na semana	42 (40,78%)
Nunca	36 (34,95%)
Dorme bem e sente-se descansado	
Sempre ou Geralmente	20 (19,42%)
Algumas Vezes	51 (49,51%)
Quase nunca ou raramente	32 (31,07%)
Utiliza drogas	
Sim	68 (66,02%)
Não	33 (32,04%)
Não informado	2 (1,94%)
Total	103 (100%)

Observa-se, na Tabela 2, que 51,4% dos estudantes já procuraram atendimento psicológico e 51,46% relataram ter tido alguma dificuldade emocional que prejudicou os estudos. Além disso, 66,02% dos estudantes indicaram o uso de drogas e 31,07% afirmaram que quase nunca ou raramente dormem bem e se sentem descansados.

Entre as alterações de saúde mental, descritas nos prontuários, que motivaram os estudantes a procurarem atendimento psiquiátrico destacaram-se ansiedade (53; 51,45%), depressão (48; 46,6%) e insônia (40; 38,83%).

### Modelo de Regressão Logística

Neste trabalho foram analisadas três variáveis aleatórias, de maneira independente, cujas respostas foram classificadas como Y=1, se o indivíduo apresentasse a característica, e Y=0, caso não apresentasse. Tais variáveis foram as alterações relacionadas à saúde mental apresentadas pelos estudantes na procura pelo atendimento psiquiátrico: ansiedade, depressão e insônia.

Cada uma das variáveis (ansiedade, depressão, insônia) foi relacionada com as seguintes variáveis explicativas: curso; ano de ingresso; idade; sexo; raça/etnia; convênio; tratamento de saúde; uso de medicamentos; antecedentes familiares; se possui problemas de pele; se possui problemas cardíacos; se possui problemas no estômago; se costuma ter diarreia; se costuma ter intestino preso; se apresenta alteração de peso; se costuma ter dor de cabeça; se já teve crise convulsiva; atendimento psicológico prévio; atendimento psiquiátrico; medicamento psiquiátrico; diagnóstico psiquiátrico; dificuldade emocional; atividade física; se dorme bem e sente-se descansado; uso de drogas; e ingestão de bebidas com cafeína, como demonstram as Tabelas 3, 4 e 5.

**Tabela 3:** Modelo final ajustado para a variável Ansiedade, São Paulo, São Paulo, Brasil, 2020-2021 (N=103)

Variáveis explicativas	Coeficiente	Valor-p
Intercepto	0,006	0,985
Realiza algum tratamento de saúde	1,074	0,076^*^
Utiliza alguma medicação	-1,216	0,029^**^
Possui algum problema de pele	0,532	0,369
Tem sofrido alteração de peso	-0,744	0,110
Já teve alguma dificuldade emocional que prejudicou os estudos	0,981	0,056^*^

* significativo a 10%;

** significativo a 5%.

**Tabela 4:** Modelo final ajustado para a variável Depressão, São Paulo, São Paulo, Brasil, 2020-2021 (N=103)

Variáveis explicativas	Coeficiente	Valor de *p*
Intercepto	-0,096	0,758
Possui problemas cardíacos	17,181	0,992
Costuma ter diarreia	1,919	0,025^**^
Possui problemas de intestino	0,685	0,140
Já teve alguma dificuldade emocional que prejudicou os estudos	-0,974	0,029^**^

* significativo a 10%;

** significativo a 5%.

**Tabela 5:** Modelo final ajustado para a variável Insônia, São Paulo, São Paulo, Brasil, 2020-2021 (N=103)

Variáveis explicativas	Coeficiente	Valor de *p*
Intercepto	-1,456	0,006^**^
Possui tratamento psicológico prévio	1,292	0,032^**^
Utiliza algum medicamento psiquiátrico	-2,401	0,041^**^
Costuma ter diarreia	1.219	0.041^**^
Costuma ter dor de cabeça	1,810	0,002^**^
Já teve alguma dificuldade emocional que prejudicou os estudos	-1,309	0,036^**^
Não pratica atividade física	0,977	0,089^*^
Pratica atividade física > 3 vezes	-0,544	0,470
Pratica atividade física diariamente	0,615	0,487

* significativo a 10%;

** significativo a 5%.

Observa-se que estudantes que afirmaram já terem tido alguma dificuldade emocional que prejudicou seus estudos (valor de *p* = 0,056) apresentam maior probabilidade de desenvolver ansiedade durante a graduação, quando comparados aos que negaram tal dificuldade emocional. Aqueles que realizaram algum tratamento de saúde apresentam maior probabilidade (valor de *p* = 0,076) de desenvolver ansiedade. Por outro lado, alunos que utilizam algum tipo de medicamento têm menor probabilidade (p = 0,029) de apresentar ansiedade.

Os estudantes que costumam ter diarreia (p = 0,025) possuem maior probabilidade de desenvolver depressão, quando comparados aos que negaram tal sintoma físico. Por outro lado, o universitário que afirmou ter tido alguma dificuldade emocional que prejudicou seus estudos (p = 0,029) tem menor probabilidade de apresentar depressão.

Diarreia (p = 0,041) e dor de cabeça (p = 0,002) aparecem como sintomas físicos relacionados à insônia. Estudantes que apresentam tais sintomas têm maior probabilidade de ter insônia, enquanto aqueles que utilizam medicamentos psiquiátricos (p = 0,041) ou possuem dificuldade emocional (p = 0,036) apresentam menor probabilidade.

Além disso, universitários que afirmam já terem feito tratamento psicológico prévio (p = 0,032) apresentam maior probabilidade de desenvolver insônia quando comparados aos que negam. Em relação à prática de atividade física, estudantes que não se exercitam (p = 0,089) possuem maior probabilidade de apresentar insônia comparados àqueles que praticam atividade física ao menos três vezes por semana.

## DISCUSSÃO

O presente estudo permitiu caracterizar o perfil sociodemográfico e psicológico de estudantes de graduação da área da saúde que procuraram atendimento psiquiátrico, além de analisar os fatores predisponentes para alterações de saúde mental.

O gênero feminino mostrou-se prevalente quando comparado ao masculino, corroborando achados na literatura que revelam a maior incidência de transtornos mentais em mulheres, como depressão, ansiedade e tentativas de suicídio^(16,17)^. Segundo a Organização Mundial da Saúde (OMS), a prevalência de ansiedade entre mulheres do continente americano é de 7,7%^(18)^. A violência de gênero, somada ao acúmulo de múltiplas funções, como vida acadêmica, sociofamiliar e econômica, são fatores que predispõem ao adoecimento mental^(19)^.

Acadêmicos da área de saúde imergem na vivência universitária de maneira mais intensa ao entrarem em contato com doenças, mortes e sofrimento de pacientes e familiares^(20)^. Neste estudo, os cursos de Medicina e Enfermagem apresentaram, proporcionalmente, números superiores de estudantes procurando atendimento psiquiátrico, quando comparados a outros cursos de graduação, como Biomedicina, Fonoaudiologia e Tecnologias. Tal achado não é conclusivo, uma vez que os cursos de Enfermagem e Medicina oferecem um número maior de vagas anuais e, consequentemente, possuem um percentual maior de estudantes no campus do estudo. Contudo, há pesquisas que mostram o adoecimento mental como consequência do maior contato com a prática clínica e ambiente hospitalar^(21,22)^.

O consumo de drogas foi relatado pela maioria dos universitários (68; 66,02%), corroborando a literatura, que mostra altos índices do uso de álcool na universidade^(23)^. Tal consumo é considerado uma estratégia de enfrentamento de diversas condições da vida acadêmica que geram vulnerabilidade aos transtornos mentais, como exigências da formação e preocupação com a inserção no mercado de trabalho^(20)^. O adoecimento mental também pode se tornar um fator predisponente para a utilização abusiva de álcool, que apresenta correlação com o uso de drogas ilícitas, alto consumo de cigarros e aumento de comportamentos de risco, como prática sexual sem uso de preservativo e direção veicular mesmo estando embriagado^(24,25)^.

Um levantamento realizado em 2016 pela OMS demonstrou que 26,4 milhões de pessoas no mundo sofrem com transtornos de ansiedade^(18)^. Esse transtorno foi a principal queixa dos estudantes que procuraram atendimento psiquiátrico na universidade. De 103 estudantes, 53 (51,45%) declararam sentirem-se ansiosos, estando de acordo com outras pesquisas que mostram índices expressivos de ansiedade entre os universitários^(26,27)^.

A dificuldade emocional esteve presente na maioria dos universitários; cerca de 53 (51,46%) alunos declararam alguma dificuldade emocional que tenha prejudicado seus estudos. Dados da V Pesquisa Nacional de Perfil Socioeconômico e Cultural de 2018, realizada pela ANDIFES, corroboram os resultados apresentados: 83,5% dos estudantes em diferentes instituições federais informaram já ter tido dificuldade emocional que tenha prejudicado sua vida acadêmica, tais como ansiedade, tristeza, timidez, pânico e insônia^(14)^.

Em contrapartida, no presente estudo, a dificuldade emocional apresentou-se como fator protetivo em relação à depressão e insônia. Tal achado contradiz a literatura, que demonstra a interferência da dificuldade emocional no rendimento acadêmico e na qualidade de vida, podendo ocasionar abandono da universidade, estresse, raiva, tristeza e desânimo, situações predisponentes ao adoecimento mental^(28,29)^.

Ademais, estudantes que afirmaram já ter tido tal dificuldade emocional apresentam maior probabilidade de desenvolver ansiedade. A exaustão emocional, como resultado da despersonalização dos universitários através de cobranças excessivas, carga horária intensa, elevada quantidade de avaliações, além de conflitos interpessoais com colegas de curso e docentes, é um facilitador para o desenvolvimento de ansiedade^(30)^.

O estresse está presente significativamente entre universitários^(31)^. Uma das principais respostas protetivas fisiológicas do corpo humano ao estresse é a liberação do hormônio glicocorticoide cortisol pelo eixo Hipotálamo-Pituitária-Adrenal, do sistema neuroendócrino^(32,33)^. Contudo, a elevada liberação de cortisol durante um longo período pode resultar em significativas alterações metabólicas, como diabetes mellitus tipo 2, hipertensão arterial e obesidade^(33)^. Além disso, o estresse crônico pode resultar no aparecimento da síndrome de Burnout, caracterizada pela exaustão física e mental^(34)^.

Na presente pesquisa, estudantes que afirmaram realizar algum tratamento de saúde têm maior probabilidade de apresentar ansiedade. O adoecimento físico entre os estudantes universitários também é um fator predisponente à presença de transtorno mental^(35,36)^.

De acordo com a ANDIFES, cerca de 23,2% dos universitários de Instituições Federais de Ensino Superior procuram serviços médicos para realizar algum tratamento específico, e 32,4% dos estudantes já procuraram atendimento psicológico^(14)^. Tais resultados condizem com a presente pesquisa, na qual 22,33% dos universitários atendidos realizam algum tratamento de saúde. Quanto ao tratamento psicológico, evidenciou-se um índice superior, com cerca de 51,4% dos estudantes referindo a procura por esse tipo de atendimento.

O atendimento psiquiátrico prévio foi constatado em 24,27% dos alunos, e o uso de psicotrópicos em 13,59%, mostrando maior índice em comparação a outros estudantes de instituições federais^(14)^.

Em relação ao consumo de medicações, 38 (36,8%) estudantes relataram estar utilizando algum medicamento, apresentando menor índice em comparação com uma pesquisa realizada em uma universidade do Planalto Norte Catarinense, na qual 234 alunos (57,2%) estavam utilizando alguma medicação^(37)^. Além disso, segundo o presente estudo, tomar alguma medicação é um fator protetivo em relação ao desenvolvimento de ansiedade, concordando com outros estudos que mostram que a busca pela saúde, autopercepção e reequilíbrio emocional diminuem fatores predisponentes ao adoecimento mental^(38,39)^. Contudo, o uso indiscriminado de medicações entre universitários pode levar a reações adversas, intoxicações e dependência^(39)^.

Os sintomas depressivos representam o segundo lugar entre os motivos mais prevalentes na busca pelo atendimento psiquiátrico. Isso corrobora com estudos que apontam o descontentamento com o curso, a competição no ambiente acadêmico, a alta exigência durante avaliações, a diminuição do apoio familiar, a baixa quantidade de sono, a solidão e a falta de lazer como fatores predisponentes para o desenvolvimento de depressão^(11,40)^. Como consequência, há diminuição da capacidade de memorização, motivação e raciocínio, interferindo no desempenho acadêmico^(40)^. O diagnóstico e tratamento precoce são de extrema relevância, visto que previnem comportamentos de risco e ideação suicida entre estudantes universitários^(40)^.

A associação entre sintomas gastrointestinais e transtornos mentais também foi resultado desta pesquisa. Estudantes que afirmaram ter diarreia apresentam maior probabilidade de desenvolver sintomas de depressão e, também, insônia. Tal achado concorda com a literatura, que mostra que o estresse interfere no funcionamento dos sistemas do organismo humano, comprometendo componentes celulares e desencadeando doenças, como a Síndrome do Intestino Irritável^(41)^. Ademais, uma revisão integrativa da literatura relacionada ao Índice de Qualidade do Sono de Pittsburgh em estudantes de medicina mostrou alta prevalência de má qualidade de sono entre alunos com Síndrome do Intestino Irritável^(42)^.

A dor de cabeça esteve presente em quase metade dos estudantes que procuraram atendimento na universidade. Tais universitários apresentam maior probabilidade de desenvolver insônia, concordando com a literatura^(43)^. O sono apresenta importante função neuronal, interferindo no funcionamento cognitivo^(44)^. A disfunção do ciclo sono-vigília provoca baixo rendimento acadêmico, interferindo no desenvolvimento profissional dos estudantes^(45)^. Dessa maneira, a insônia foi a terceira alteração mais relatada no atendimento psiquiátrico, corroborando achados de outros estudos^(46,47)^.

A prática de exercício físico demonstrou ser um fator protetivo em relação à insônia. Estudantes que não praticam atividade física apresentam maior probabilidade de desenvolver insônia quando comparados aos que praticam pelo menos três vezes na semana. Ao se exercitar, o corpo entra em um processo catabólico que precisa ser recompensado, posteriormente, por um processo anabólico, resultando em maior relaxamento e vontade de dormir^(48)^. Além disso, o exercício físico proporciona maior regulação emocional, diminuindo o estresse e aumentando a qualidade do sono^(46)^.

### Limitações do estudo

A pesquisa foi realizada em um campus que concentra apenas cursos da área da saúde, portanto, não permite determinar o padrão de adoecimento psíquico considerando diferentes perfis de cursos e estudantes. Além disso, o instrumento avaliado foi construído para a coleta de informações do estudante com a finalidade de qualificar necessidades gerais e, assim, não aprofunda questões que seriam relevantes para processos de adoecimento em saúde mental.

### Contribuições para a Área da Saúde

Os resultados deste estudo podem fundamentar estratégias para melhorar a qualidade de vida e o sucesso acadêmico dos estudantes universitários.

## CONCLUSÕES

Estudantes do gênero feminino compuseram a maioria dos que procuraram atendimento psiquiátrico em um serviço de atendimento a universitários. Sintomas de ansiedade, depressão e insônia foram os principais motivos para a procura de atendimento. A análise inferencial encontrou maior probabilidade estatística para sintomas de ansiedade nos estudantes que afirmaram ter dificuldade emocional e tratamento de saúde prévio, maior probabilidade de sintomas de depressão em estudantes que apresentavam queixa de diarreia e maior probabilidade de insônia em estudantes com queixa de diarreia, dor de cabeça e que não praticam atividade física.

A vivência universitária coincide com o período de mudanças para a fase adulta e o aumento de responsabilidades para a maioria dos estudantes. Criam-se expectativas de rendimento acadêmico e sucesso profissional, além da influência de fatores biográficos e de gênero, raça/etnia e classe social que podem interferir nos processos de saúde-adoecimento mental.
